# What It Means to Be Guinea Worm Free: An Insider’s Account from Ghana’s Northern Region

**DOI:** 10.4269/ajtmh.17-0558

**Published:** 2018-03-19

**Authors:** Adam J. Weiss, Torben Vestergaard Frandsen, Ernesto Ruiz-Tiben, Donald R. Hopkins, Franklin Aseidu-Bekoe, David Agyemang

**Affiliations:** 1 The Carter Center, Atlanta, Georgia;; 2 Vestergaard, Lausanne, Switzerland;; 3 Ministry of Health, Accra, Ghana;; 4 Sightsavers, Accra, Ghana

## Abstract

Despite several periods of stagnating guinea worm disease (GWD) incidence in Ghana during its national eradication campaign in the 1990s and early 2000s, the last reported case of GWD was in May 2010. In July 2011, Ghana celebrated the interruption of guinea worm (GW) transmission. Although it has been established that GWD causes disability, pain, and socioeconomic hardship, there is a dearth of population-based evidence collected in post-GW–endemic countries to document the value attributed to GWD eradication by residents in formerly endemic communities. Given Ghana’s recent history of GWD and a concentrated burden of the disease in its Northern Region, a pattern which remained true through to the final stage of the eradication campaign, seven villages in the Northern Region were targeted for a retrospective, cross-sectional study to detail the perceptions, attitudes, and beliefs about the impact of eradication of GWD in northern Ghana. The study revealed that respondents from the sampled communities felt GW eradication improved their socioeconomic conditions, as the impact of infection prohibited the pursuit of individual and social advancement. The value residents placed on the absence of GWD highlights both the impact infection had on the pursuit of social and economic advancement and the newfound ability to be disease-free and productive. Of the 143 respondents, 133 had GWD in the past and were incapacitated for an average of 6 weeks annually per GW infection, with each infected person affected nearly four times in his or her lifetime.

## INTRODUCTION

*Dracunculus medinensis*, commonly referred to as guinea worm (GW), is a nematode parasite that typically affects only human beings.^1,2^ Also known as the *fiery serpent* because of the generalized burning sensation one feels when the worm is about to emerge, GW is referenced in the bible and has been recovered from mummies in Ancient Egypt nearly 3,000 years old.^1^ The genesis of the idea to eradicate guinea worm disease (GWD) had its origin at the Centers for Disease Control and Prevention in 1980 following the success of the smallpox eradication campaign.^3^

In 1986, former U.S. President Jimmy Carter agreed to become the lead advocate for eradication of GWD and for the Carter Center to provide technical and financial assistance to countries with endemic GWD, including Ghana. The Carter Center efforts to assist Ghana with its national effort to eliminate transmission of GWD in Ghana were fully supported by the then President, Flight Lieutenant Jerry Rawlings, who inaugurated the Ghana Guinea Worm Eradication Program (GGWEP) in 1988.^4^ The next year the program detected 179,556 cases of GWD.^4^ This was the second highest recorded incidence of GWD only in Nigeria.^4,5^ Most of the cases were found in the Northern Region, a pattern which remained true until the end of the campaign.^6^ Nearly all of the socioeconomic and health indicators in the Northern Region ranked between eighth and 10th of the 10 regions and thus lower than the national average.^7^ Residents of the Northern Region are primarily subsistence farmers and practice small-scale animal husbandry. The region experienced repeated internal conflicts throughout the mid-1990s, including the highly referenced “Guinea Fowl War,” which led to an exodus of health workers, including U.S. Peace Corps volunteers, and a reduction in the provision of health services, including interventions to eradicate GWD.^8,9^ These challenges, together with a long dry season (November–April), difficulties in extracting safe drinking water from underground sources, and the creation of large impoundments to provide water for farmers and their livestock and also for humans, provided suitable conditions for the transmission of GW.^10^

Ghana’s last reported case of GWD was in May 2010.^6^ Having successfully observed 14 months of zero case reports in the presence of an active village-based surveillance system, Ghana celebrated the interruption of transmission in July 2011.^11^ Following 3 years of zero case reports, the GGWEP submitted a comprehensive country report on the history of GWD in the country, including the eradication campaign, to the International Commission for the Certification of Dracunculiasis Eradication (ICCDE) requesting approval to be certified free of GWD. In July 2014, the ICCDE sent an independent International Certification Team (ICT) to verify Ghana’s claim of having interrupted transmission and, following verification by the ICT, recommended Ghana be certified free of GWD. In January 2015, Ghana was granted certification by the World Health Organization.^12^

Formative literature about the culture and people of the Northern Region, including perceptions about western medicine and several studies elaborating the financial impact of GWD in Ghana and Nigeria, exist.^13–15^ Although myriad research has been conducted on GW’s biology, life cycle, and epidemiology since the late 1800s, there is no population-based evidence documenting the value attributed to GWD eradication by residents in endemic communities, either in a pre- or post-eradication environment.^2,13,15–22^ This study was designed to understand the attitudes and perceptions of Ghanaians after the eradication of GWD to capture the voices of those affected by the disease.

## METHODS

### Retrospective, cross-sectional study.

The study was retrospective and cross-sectional as it detailed perceptions, attitudes, and beliefs about the impact of eradication of GWD in northern Ghana. Because of the dearth of literature about attitudes toward GWD in northern Ghana, house-to-house surveys and focus group discussions (FGDs) were conducted. Furthermore, the investigators used a concurrent study strategy so that the survey tools captured relevant demographic data and detailed direct and indirect experiences with GWD simultaneously.^23^ Before implementation, a pilot test of the survey instrument was conducted in Tamale town, capital of the Northern Region. The sample represented the same demographic targeted in the study, and the pilot test confirmed the reliability and validity of the survey tools. In addition, the pilot test provided insight into the logistical planning necessary to complete the study.

### Village selection.

**Figure 1.**
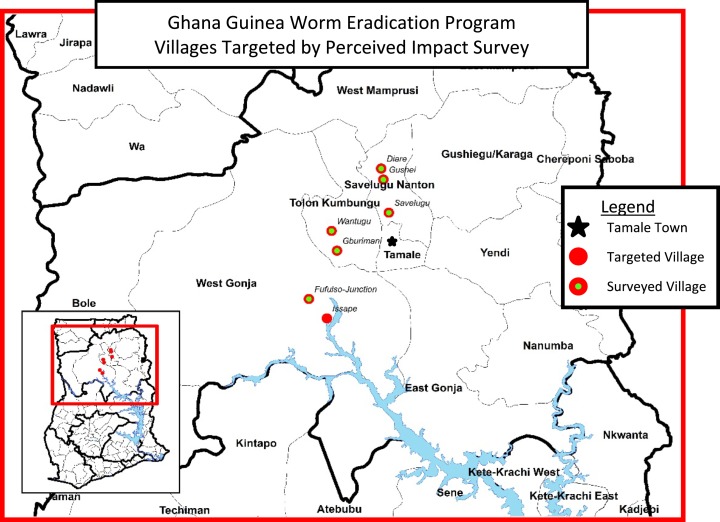
Map of communities targeted and surveyed. This figure appears in color at www.ajtmh.org.

Because of the historically high incidence of GWD in the Northern Region and the occurrence of GWD in peri-urban settings, the study targeted both urban and rural communities to generate an understanding of perceptions in both environments. The peri-urban towns of Savelugu, Diare, and Fufulso Junction were surveyed in addition to the more remote villages of Gburimani, Wantugu, Issape, and Gushie. The communities were all in the Northern Region and represented both the Dagomba and Gonja ethnic groups, the two ethnic groups most heavily affected by GWD in Ghana (Figure 1).

The study targeted a minimum of 30 household interviews and five FDGs in each of the three towns (Savelugu, Diare, and Fufulso Junction). In the four smaller communities (Gburimani, Wantugu, Issape, and Gushie), a minimum of 5–20 interviews and two to three focus groups were targeted. In the communities where schools were present, at least one FGD was targeted in a school setting.

A staff member of the Ghana Health Service provided translation to and from English into Dagbani and Gonja for the interviews. A handheld tablet was used to collect the quantitative data and paper questionnaires were used to record responses to open-ended questions. To ensure the accuracy of the transcription, interviews were also recorded and transcribed daily.

### Household and participant selection.

Households were selected randomly. In each household, the head of the household (or representative) was selected to be interviewed and was read the verbal consent form to ensure the respondent was adequately informed of the nature and substance of the interview. If consent was provided, the investigator further inquired if the interview could be digitally recorded. On approval, a private location was identified to ensure privacy and prevent potential bias from bystanders. After completing the first interview, a second interview was conducted with another member of the same household.

After surveying the household, the investigator spun a bottle to identify the next household. To minimize the clustering of households, the interval between houses was determined based on the size of the village. In smaller villages (< 500 population), the first house in the direction of the bottle was visited. In large peri-urban settings (e.g., Savelugu), the third house in the direction of the bottle was selected until the prescribed number of sample houses was met. In villages with populations between 500 and 4,000, 5–20 household interviews were conducted (Table 1) and in larger villages, 4,001–44,000, at least 30 households were selected.

**Table 1:** Communities targeted and implemented surveys

Village name	Population (2009)	No. of households	No. of households targeted	No. of households surveyed	No. of focus groups targeted	No. of focus groups surveyed
Savelugu	43,234	4,323	30	30	10	3
Diare	13,836	1,383	30	30	7	3
Fufulso junction	5,524	552	30	32	5	3
Gburimani	2,525	252	10	21	2	3
Wantugu	4,426	442	20	20	3	2
Gushei	1,270	127	7	10	2	2
Issape	520	52	5	0	2	0
Tamale (schools only)	360,579	13,694	0	0	2	2
Total	71,335	7,131	132	143	33	18

### Focus group selection.

In addition to household interviews, FGDs were conducted to ensure that perceptions across demographic groups were obtained. Study criteria included selection across three age groups (5–18, 19–35, and older than 36 years of age), principal occupation (farmers, housewives, and traders), both genders, and, where available, students. Focus groups were organized with students at schools and with adults at social and market gathering sites. During group discussions, the investigator ascribed comments by gender and approximate age of the respondent to simplify the summation of responses. At least three focus groups were held in each village (e.g., school, market, and social gathering).

The investigator met with district health officials and community chiefs to satisfy community entry protocol and was consistent in the administration of each question to preserve the integrity of the survey and ensure consistency throughout implementation.^24^ Permission to conduct and record interviews was obtained from all respondents. According to local regulations, the headmaster provided consent to discuss GWD in schools; however, general consent among the students was requested and anyone electing not to participate was not obligated to do so.

### Ethical aspects.

The Ghana Ministry of Health approved the study as operational research and, thus, required no further review. Before the study’s implementation, the project proposal and study tools were submitted to the Emory University Institutional Review Board (IRB) for review. The IRB determined that the study did not require review. All respondents provided verbal or written informed consent before being interviewed.

## RESULTS

In October 2013, a total of 143 head of household interviews were conducted in six of the seven targeted towns and villages. Issape, in the Central Gonja district, could not be visited because of inaccessibility caused by flooding after heavy rains. In addition, 18 FGDs were conducted involving 400 residents across all age groups, including two schools in Tamale town.

Of the individual respondents, 74 were male and 69 were female, with generally equitable distribution across the 16–25, 26–50, and 51+ years age groups (Table 2). Farming and housewifery were the two dominant occupations, representing 70% and 52% of the male and female respondents, respectively. Although most of the housewives also actively participated in farming activities, only their first response was analyzed as it reflects their primary occupation.

**Table 2:** Reported personal experience with GWD of 143 individual respondents

Individual respondents	Number	Number ever had GWD	Average years since Last infection of GWD (range)	Average number of GWs in lifetime (range)	Average number of times GW emerged during lifetime (range)	Average no. of weeks incapacitated per GW event (range)
All	143	133	11.9 (2–60)	7.3 (1–75)	3.9 (1–30)	5.8 (1–52)
Male	74	70	13.9 (1–60)	7.2 (1–40)	4 (1–20)	6.5 (1–52)
16–25	22	22	9.1 (2.5–22)	2.9 (1–6)	1.8 (1–3)	4.1 (1–12)
26–50	22	21	12.1 (3–30)	8 (1–35)	4.3 (1–15)	7.4 (0.5–52)
51+	30	27	19.3 (1–60)	9.4 (1–40)	5.6 (1–20)	9 (1–52)
Female	69	63	9.7 (3–45)	7.7 (1–75)	3.9 (1–30)	4.5 (1–20)
16–25	16	15	6.1 (3–10)	6.7 (1–30)	2.9 (1–10)	4.4 (1–12)
26–50	33	29	11.6 (3–40)	7.7 (1–75)	4 (1–30)	5 (2.5–20)
51+	20	19	9.6 (2–45)	7.6 (1–35)	4.1 (1–10)	4 (1–8)

GW = guinea worm; GWD = GW disease.

**Figure 2.**
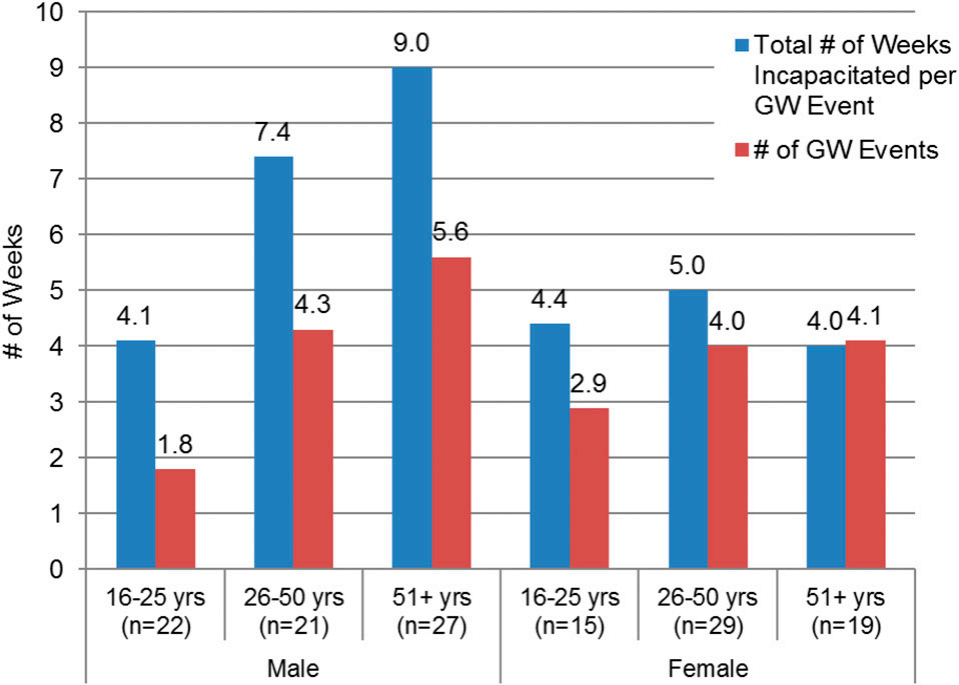
Average number of weeks respondents were incapacitated and the number of guinea worm events they experienced in their lifetime. This figure appears in color at www.ajtmh.org.

Of the 143 respondents, 133 (93%) claimed to have experienced GWD at least once in their lifetime. On average, respondents had nearly four GW events (separate occurrences of GWD, not multiple worms at one time during the course of a given year) and seven worms in their lifetime (Figure 2). Ninety-seven percent (129/133) of the infected respondents were incapacitated for an average of 6 weeks per GW infection. Several respondents indicated that they were incapacitated for an entire year because of complications resulting from secondary bacterial infections and multiple worms emerging throughout the year. On average, females reported fewer worms and shorter periods of incapacitation (Figure 2). All respondents who had GWD reported scarification at the site of worm emergence. Three percent (4/133) of the respondents indicated continued pain associated with the area where the worms emerged and 1.5% (2/133) reported persistent difficulty ambulating because of sequalae from GW infections. This is consistent with studies highlighted by Imtiaz et al.^21^

**Figure 3.**
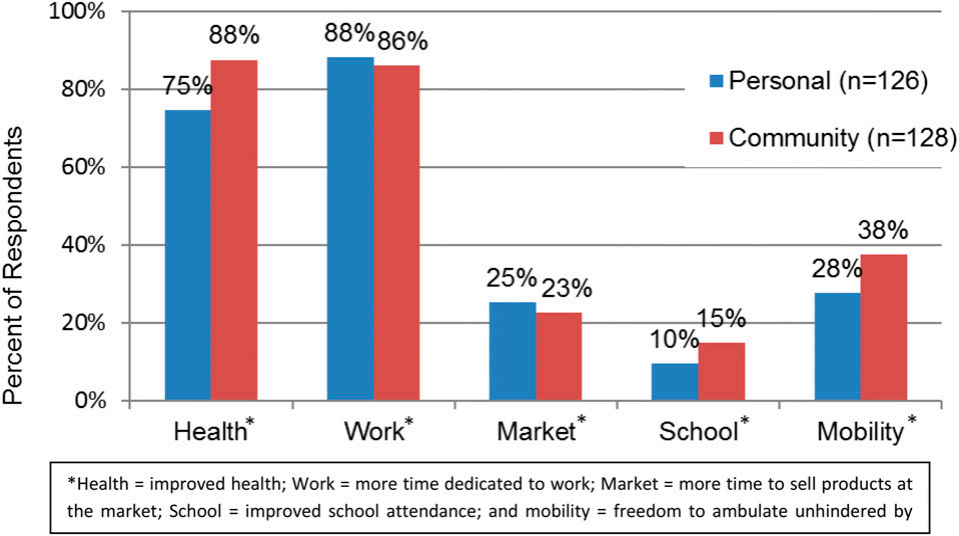
Personal and community perception of beneficial effects resulting from guinea worm disease (GWD) eradication. * Health = improved health; work = more time dedicated to work; market = more time to sell products at the market; school = improved school attendance; and mobility = freedom to ambulate unhindered by GWD. This figure appears in color at www.ajtmh.org.

All 143 household respondents indicated that the absence of GWD had changed their life, both individually and as a community. Results from open-ended questions about how and in what ways the absence of GWD impacted their lives individually revealed that between 75% and 88% of respondents believed health and work (i.e., farming, business, and household chores) improved significantly (Figure 3). Respondent no. 26 said, “our people were relieved of a spell” and that “I am now able to work in my farm to produce enough food for my children.” Of female respondents mentioning improved health, only 15% mentioned the ability to bear and rear children and care for their families. More males cited improved agricultural productivity, whereas females indicated improved health. All female respondents who cited improved market and work activity also mentioned the ability to focus more time and attention to small business and trading activities. Similarly, all the men indicated an increase in productivity from farm labor. Respondent no. 47 said he “lost many harvests because of GW and that the rainy season, for a long time, was not looked forward to because GW might come out in large numbers.” Although health and work were described as principal benefits, between 10% and 38% of respondents also noted improved mobility, market activities, and school attendance. Similar themes were identified by respondents describing life post-GW from a communal perspective, with only a slight increase in references to health and mobility improvements. There was no mention of psychological improvement, but the inability to sleep and frustration from not working were described. There was no distinguishable difference between Dagomba and Gonja respondents.

Individual interviews were triangulated with 18 FGDs (including six school focus groups). The primary themes identified during discussions among nearly 400 focus group participants were consistent with those of the individual interviews and included health, work, economics, and school performance, signaling saturation was likely achieved during individual interviews. More than 90% of the focus groups identified work as the primary activity impacted by GWD and referenced the impact as being more significant at the community level. Overall, 99.8% of respondents (542/543) expressed that GWD had a negative impact on their lives and that they had experienced improvement in their livelihood post-GW eradication. Focus Group no. 12 shared that their “children can now become professional football players and learn freely,” reflecting their perception that a future without GW was more hopeful.

### Limitations.

The methodology applied in the study was limited in several ways. Recall bias was a primary limitation, given the average 12-year gap between 2013 and respondents’ most recent infection with GW. This potentially skewed the study’s findings by over- or understating the perceived changes post-GW eradication. In addition, courtesy bias and a perception that new community programs would be provided if respondents spoke positively of the GWEP may have influenced responses. The themes addressed by respondents were sincere as they were consistent with the impact and effects associated with a physically incapacitating disease. Respondents provided accounts of their experience with GW, but there is no baseline data to compare their perceptions before the eradication of GW. Despite improved macroeconomic conditions in Ghana during the past decade, which could have also influenced respondents’ general perceptions, most respondents focused on the direct impact on their physical inputs and day-to-day life activities.

## DISCUSSION

Although interruption of GWD transmission nationwide in Ghana satisfies the definition of a public health success, this study provides an account of how the eradication of GWD was perceived in northern Ghana. This survey is the first known attempt to collect population-based evidence from residents of affected communities in a country formerly affected by endemic GWD to document residents’ perception of value attributed to GWD eradication.

The study showed that 97% of the respondents were incapacitated during the agricultural season and were unable to carry out their normal chores (laboring to produce food for their families) for an average of 6 weeks during each GW emergence event. This is also consistent with the 6–15 weeks of incapacitation reported in previous studies.^13,15,20^ The study also showed that the duration of incapacitation was longer for people older than 25 years of age, with males > 51 years and older experiencing more than five GW events and incapacitation for 9 weeks (Figure 2). This represents the adult working cohort, who, by virtue of their responsibilities, drink more unsafe water outside of the household and were thereby at a higher risk for acquiring the disease. Because farming is the main occupation for most of the respondents, incapacitation had a direct impact on agricultural productivity.^15^ A United Nations Children’s Fund (UNICEF)-supported study in Nigeria estimated that about US $20 million was lost annually in rice production alone.^25^ Furthermore, as identified by other researchers, children also missed school either because they were incapacitated by the disease or as a consequence of replacing sick relatives in agricultural activities.^26^ Nearly all respondents specifically mentioned that GWD resulted in lost crop production, a loss of dignity, and economic and social hardship not only in the last decade but also as far back as their oral histories could recount.

The study sample did not allow the comparison of results between the Dagomba and Gonja ethnic groups, two groups heavily affected by GWD in Ghana, as the targeted communities were in Dagomba cultural areas. However, based on the available sample of Gonja respondents, there was no indication that they perceived the absence of GWD differently than the Dagomba.

The anticipated benefits of a successful GWEP are widely understood. However, little attention has been given to how local populations perceive the disease. One study in northern Ghana, among the Anufo ethnic group, looked at disease classification in terms of how GW was ranked against other more virulent diseases,^14^ though the study did not address how the existence or absence of GW would impact their lives. As public health programs proceed through their own life cycle, the responsibility to remain focused on the beneficiaries does not subside. This requires following up with beneficiaries even after the supposed success of the program to reinforce the value of the lessons learned during the program.

Since the beginning of the GGWEP, the endemic regions of Ghana have not benefitted from significant infrastructural development.^7^ Apart from what the GGWEP did to garner support for safe water development from partners, including UNICEF, the European Union, the Conrad N. Hilton Foundation, and Rotary International to name a few, direct government investment was meager until near the end of the program. The failure to improve the standard of living across the country has required the investment of millions of dollars to deal with GWD and other neglected tropical diseases (NTDs) that might have otherwise been unnecessary.

Although this study highlights Ghanaians’ perceptions about GWD eradication, additional studies should consider evaluating the impact of the indirect benefits, such as the provision of safe water and health education that formerly endemic communities received as a result of the GGWEP in formerly GW endemic areas. The GGWEP, in particular, was able to garner substantial investment toward the provision of safe water in endemic areas, yet the level of development has been slow and uneven. Public health economists have reconfirmed the cost-benefit of pursuing eradication of GWD but more should be done to determine which programmatic elements have led to the cost-effectiveness.^27^ The documentation of an insider’s view of what eradication of GWD has meant to the people of Ghana may help policymakers and funders uphold commitments outlined in the London Declaration on NTDs.^28^

As the final chapter of the global GW eradication campaign nears, the importance of understanding the perceived value of the absence of the disease among previously affected residents cannot be overstated. Thankfully, GW will become a forgotten disease, but the lessons for public health remain profound. Documenting the value of removing a disease or problem, in quantitative and qualitative terms, will help future public health programs plan interventions with new insight into the very population targeted. There is no final public health program, and each must learn from those that have come and gone before it, just as the GWEP was born out of the success of the smallpox eradication campaign, so too another will come after GW is gone.
